# Fluorescent Nanoprobes Dedicated to *in Vivo* Imaging: From Preclinical Validations to Clinical Translation

**DOI:** 10.3390/molecules17055564

**Published:** 2012-05-10

**Authors:** Juliette Mérian, Julien Gravier, Fabrice Navarro, Isabelle Texier

**Affiliations:** Département microTechnologies pour la Biologie et la Santé CEA-LETI, Minatec, 17 rue des Martyrs, 38045 Grenoble cedex, France; Email: juliette.merian@cea.fr (J.M.)

**Keywords:** fluorescence imaging, nanoprobes, *in vivo* imaging, nanoparticles, organic dyes, quantum dots, contrast agents, biodistribution, lymph node mapping

## Abstract

With the fast development, in the last ten years, of a large choice of set-ups dedicated to routine *in vivo* measurements in rodents, fluorescence imaging techniques are becoming essential tools in preclinical studies. Human clinical uses for diagnostic and image-guided surgery are also emerging. In comparison to low-molecular weight organic dyes, the use of fluorescent nanoprobes can improve both the signal sensitivity (better *in vivo* optical properties) and the fluorescence biodistribution (passive “nano” uptake in tumours for instance). A wide range of fluorescent nanoprobes have been designed and tested in preclinical studies for the last few years. They will be reviewed and discussed considering the obstacles that need to be overcome for their potential everyday use in clinics. The conjugation of fluorescence imaging with the benefits of nanotechnology should open the way to new medical applications in the near future.

## 1. Introduction

From the first anatomical charts to the most recent development of Magnetic Resonance Imaging (MRI) or Positron Emission Tomography (PET), setting into images what is normally unseen within living systems has been a major issue for the understanding of biological processes [1,2,3]. With the exception of MRI, which relies on magnetic properties of atom nuclei with half-integer spin, most of the imaging modalities rely on photons with wavelengths ranging from gamma emission to infrared. Any photon has specific interactions with tissues or bones that will define its uses and drawbacks. On the one hand, high-energy photons such as gamma emission or X-rays can go through the tissues with low interactions, making them suitable for deep tissue or whole body imaging, while being potentially harmful because of the reactions they can trigger within cells. Their use is thus strictly restricted to dedicated areas and under the supervision of trained personnel. On the other hand, photons in the visible and infrared wavelengths do not penetrate deeply within organic tissues, but they cause no or little damage to cells. Moreover, light sources and imaging apparatus are widespread, relatively cheap, their handling requires moderate levels of training and protection, and does not generate radioactive wastes.

Contrary to tissue auto-fluorescence recording, near-infrared/visible fluorescence is an optical imaging modality that relies on the injection of an exogenous probe that will emit light when excited with suitable wavelength. This method can be used from the macroscopic to the microscopic range, goes as further as sub-cellular resolution, and is highly sensitive. Targeted fluorescent probes can be designed to specifically mark and visualize different biological targets, which can be imaged with high contrast using the appropriate set of optical filters to minimize auto-fluorescence contribution of the surrounding tissues. Fluorescence is particularly suitable for *in vitro* and superficial *in vivo* imaging applications. However, fluorescence imaging using the near infrared range is also now routinely used for whole body imaging of small animals in preclinical studies [4,5,6,7], and the first human clinical trials have been performed in 2008/2009 for sentinel lymph node mapping in oncology by different groups [8,9,10,11,12]. The next challenge to address is to pursue the development of fluorescence imaging techniques in medical applications. Since the instrumentation tools now exist [13,14,15,16,17,18], the main blocking point to overcome for the expansion of clinical trials remains the availability of performing fluorescent tracers [19,20]. Nanotechnologies could facilitate their design by allowing modular and flexible constructions. This review will focus on the design of near-infrared fluorescent agents based on nanostructures, their benefits for *in vivo* imaging, and the coming challenges for their clinical translation.

## 2. Clinical Applications of *in Vivo* Fluorescence Imaging Probes and Associated Constraints

### 2.1. The Near-Infrared Window

**Figure 1:**
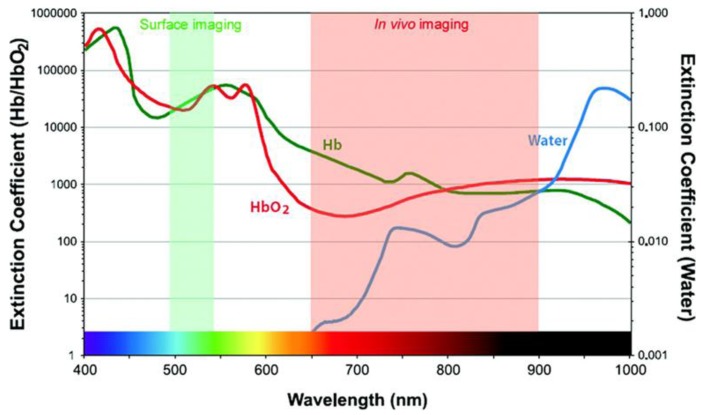
Definition of the optical window for *in vivo* imaging. Reprinted from Kobayashi, H.; Ogawa, M.; Alford, R.; Choyke, P.L.; Urano, Y. New strategies for fluorescent probe design in medical diagnostic imaging. *Chem. Rev.* **2010**, *110*, 2620–2640 [20]. Copyright (2010) American Chemical Society.

One of the driving issues for the development of new fluorescence imaging probes dedicated to clinics is the necessity for near-infrared tracers providing high signal-to-background ratio. This requires both targeting abilities of the probes to accumulate specifically in cells to be labelled, while being cleared from surrounding tissues, and highly bright fluorophores absorbing and emitting in the near infrared range. *In vivo* imaging is indeed limited by the scattering and absorption of light by tissue components, essentially blood (oxy- and deoxyhemoglobin, respectively HbO_2_ and Hb) and water. The spectral properties of these fluids thus define a narrow ‘optical window’, between 650 and 900 nm (Figure 1), where light is able to penetrate deeper, typically a few centimetres depth. Moreover, tissue auto-fluorescence is also reduced in the near infrared domain in comparison to the visible range.

Therefore, contrary to other modalities such as MRI and nuclear imaging for which complete body scans can be performed, the spectrum of fluorescence imaging applications in clinics could appear reduced. However, due to the cheap and easy handling of fluorescence tools (absence of radioactivity for instance), new applications have emerged, which could not be so easily implemented with other modalities for reasons of cost and apparatus flexibility. This is particularly the case of applications for which superficial imaging is needed. Up to now, the most investigated clinic applications are in the field of image-guided surgery [18]. In the future, other applications in the field of diagnostics, especially thanks to endoscopic instruments, can be envisioned [18,21].

### 2.2. Image-Guided Removal of Sentinel Lymph Node

**Figure 2:**
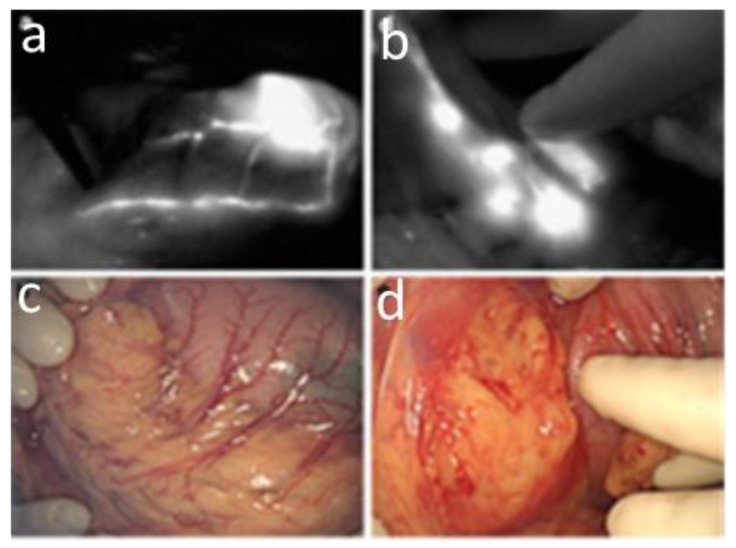
Sentinel lymph node mapping in gastric cancer surgery using fluorescence imaging. Fluorescence (**a,b**) and video (**c,d**) images showing fine lymphatic vessels (**a,c**) and the succession of lymph nodes (**b,d**). Reproduced from Miyashiro, I.; Miyoshi, N.; Hiratsuka, M.; Kishi, K.; Yamada, T.; Ohue, M.; Ohigashi, H.; Yano, M.; Ishikawa, O.; Imaoka, S. Detection of sentinel node in gastric cancer surgery by indocyanine green fluorescence imaging: comparison with infrared imaging. *Ann. Surg. Oncol.* **2008**, *15*, 1640–1643 [8], with kind permission from Springer Science+business Media.

**Figure 3:**
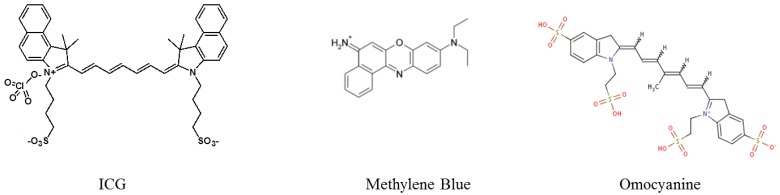
Near infrared fluorescent dyes that have been used in clinical trials until now.

The first clinical trials in the field of oncology using fluorescence imaging were published in 2008/2009 by Frangioni’s and Sevick-Muraca’s groups using home-made imaging systems [10,12], and in three Japanese teams using the Photodynamic Eye developed by Hamamatsu [8,9,11] (Figure 2). They were focused on the use of fluorescence as an image-guiding intraoperative method for sentinel lymph node resection. Precious insights about the possibility of metastatic progression can be obtained by removing the sentinel lymph node, first lymph node draining the tumour, and analysing it to determine the presence or absence of malignant cells [19]. Indocyanine Green (ICG, emission ≈ 800 nm, Figure 3), presently the only near infrared fluorescent dye with absorbing/emitting wavelengths > 700 nm to be approved by the FDA (US Food and Drug Administration) for human use, was used as tracer in these first clinical trials. The dye was locally injected intra- or sub-dermal at the tumour site. 

This first clinical application of fluorescence imaging certainly emerged for several reasons. From the instrumentation point of view, the selected applications (breast cancer, gastric cancer surgery) expose the tissues during intervention, therefore being examples of surface or low-depth image-guided surgery (typically 1 to 2 cm depth of tissues explored), turning down the challenges of deep fluorescence imaging. From the tracer point of view, the very short lifetime of ICG in blood (2 to 4 min plasma half-life, followed by a slower clearance rate once bound to plasma proteins [22]) limits its use for applications requiring systemic injection and substantial blood concentration of the dye on the long term, as required for instance for tumour fluorophore uptake. On the contrary, the lipophilic nature of ICG favours its lymphatic drainage following intradermal or sub-dermal injection at the tumour site, and its subsequent labelling of the lymph nodes. 

This example underlines that fluorescent tracers dedicated to human clinical use should respond to specific requirements, which may vary according to the applications. In the case of fluorescence-guided sentinel lymph node resection, the tracer should display no toxicity following local injection at the imaging doses, be efficiently drained by the lymphatic system, and be retained in the lymph node to be removed. 

The nanometer size (typically less than 50 nm diameter) has been demonstrated to be particularly suitable for lymph node imaging, since nano-objects, especially lipid-based ones [23,24], are preferentially retained in lymph nodes (demonstrated in different animal models, from rodents to pig [19,25,26,27,28]), in contrast to low molecular weight fluorescent dyes, more easily washed and eliminated. The adsorption of ICG on Human Serum Albumin (HSA), forming nanocolloids of ≈7 nm diameter, has been reported to significantly prolong the fluorescence signal in the sentinel lymph node of breast cancer patients [12]. Another case study, however, claims that the dye conjugation with the protein does not bring any significant benefit (time of intervention: 15 minutes following injection) [29]. Yet, sentinel lymph node mapping is one of the most studied applications concerning fluorescent nanotracers, and will certainly constitute one of the first clinical fluorescence imaging applications benefiting of improvement brought by nanotechnologies on the design of tracers.

### 2.2. Other Clinical Applications

Few other applications of fluorescence imaging have been explored in human clinical trials until now. ICG has been used since the 1970s for retinal angiography, cardiac output and hepatic function assessment [30]. More recently, it was employed as an intra-operative staining fluorophore in surgery, to image vascular network [31,32], bile ducts [33], and the demarcations of liver segments and sub-segments [34]. Methylene blue (emission ≈ 700 nm, Figure 3), another dye mainly used since now for its colour staining properties, is also slightly fluorescent in the near infrared domain [18]. It has been used for years as a tissue staining dye for visible imaging and other clinical applications, and is FDA approved for some indications [18]. Methylene blue recently allowed the identification of bile ducts and ureters during fluorescence-guided surgery [33,35]. However, it displays even poorer optical properties than ICG in terms of extinction coefficient and fluorescence quantum yield, and requires shorter excitation wavelengths, less favourable to use for deep imaging. The potentialities of ICG for dynamic fluorescence imaging to assess the lymphatic architecture and transport in healthy and lymphedema-diagnosed subjects was also explored [36]. ICG was also used to assess the presence of breast tumours by fluorescence imaging methods associated with pharmacokinetic modelisation of the dye biodistribution [37]. Another fluorescent dye, Omocianine (Bayer Shering Pharma, emission > 750 nm, Figure 3), was tested for the detection of malignant breast lesions in women suspected of breast cancer [38,39].

All these very recent studies (<4 years) demonstrate that clinical fluorescence imaging is exponentially growing, and will soon be implanted in more surgery rooms, and explored in other applications. However, they also evidence the poor availability of approved near infrared tracers (only ICG is validated by the FDA for fluorescence imaging, whereas methylene blue and Omocianine have been accepted in punctual clinical trials, Figure 3). Hence, there is a large avenue for the development of more efficient fluorescent probes. In the next paragraph, specific advantages that could be brought by nanotechnologies on the design of fluorescent tracers for clinical imaging will be discussed, whereas in Section 4, main nanosystems reported in the literature, currently undergoing preclinical studies, will be described. 

## 3. The Benefits of Nanotechnologies

### 3.1. Limitations of Low Molecular Weight Fluorophores for *in Vivo* Fluorescence Imaging

**Figure 4:**
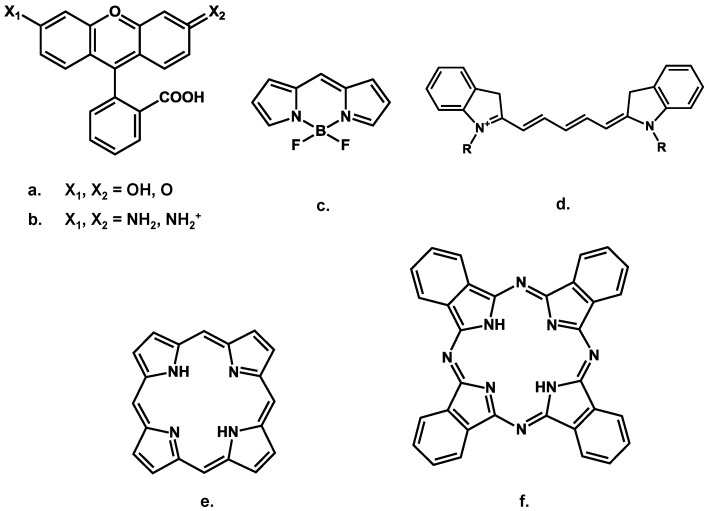
Basic chemical structures of (**a**) fluoresceins, (**b**) rhodamines, (**c**) bodipys, (**d**) indocyanines, (**e**) porphyrines and (**f**) phthalocyanines.

Low molecular weight fluorescent dyes (typically molecular weight up to ≈1,500 Da for near infrared fluorophores) are currently the most widespread type of tracers. This is mainly due to their well-defined chemical structure, which can be tailored according to the desired optical and chemical properties, and the very large commercial offer. The most common organic fluorophore families include rhodamines, BODIPY, indocyanines, porphyrines and phthalocyanines, but this list is far from comprehensive [40]. Each of the basic structures presented in Figure 4 has been modified by functional groups in order to adjust emission wavelength, enhance photostability, change hydrophobic/hydrophilic balance or to enable conjugation with targeting moieties. In the near-infrared domain (600–900 nm), mainly cyanine, bodipy, porphyrine and phtalocyanine structures are explored [41].

However, small organic fluorescent dyes present drawbacks that arise especially when it comes to *in vivo* applications. Firstly, due to their aromatic structure, these molecules are generally poorly soluble in aqueous medium, thus poorly bioavailable. This limited water solubility issue is particularly stressed for near infrared dyes, for which the addition of pi-conjugated bounds increase the molecule hydrophobicity. Chemical modifications to improve water solubility by adding polar groups, as for instance sulfonate [42,43] or saccharide [44,45] functions, have been proposed. Another drawback is: the more red-shifted the emission of a dye is, the lower its fluorescence quantum yield, which generally results in reduced brightness. Indeed, due to the increasing number of bounds involved in the pi-conjugated system, infrared emitting dyes possess a higher degree of vibrations, leading to an increasing number of non-radiative decay pathways. 

As already illustrated with the ICG dye, a second driving issue in the development of potent fluorophore for *in vivo* imaging applications, is their very fast body clearance, by kidneys and urine excretion for highly hydrophilic compounds (such as fluorescein), by liver and bile excretion for more lipophilic molecules (such as ICG). If this fast clearance can be advantageous to limit dye toxicity, or for specific applications such as for instance repetitive vascular imaging, it also limits the temporal window in which imaging can be performed, and the list of organs in which the fluorescent tracer can be distributed. A “targeting” moiety can be conjugated to the fluorescent tracer to improve the localization and binding of the dye in the area to image, and to modify its pharmacokinetics. The targeting moiety can be for instance an antibody, protein or peptide, an oligonucleotide, a saccharide, or any other molecular template known for its specific affinity for a cellular compartment, cellular receptor, biological fluid or tissue. The use of nanotemplates, where the fluorescent dye can be encapsulated, adsorbed, or grafted on the surface, constitutes another strategy to modify low molecular weight fluorophore biodistribution. In this strategy however, caution should be paid on the potential toxicity issues raised by the new biodistribution, especially uptake and prolonged retention in liver and spleen, as further discussed below.

### 3.2. What Can Nanotechnologies Bring in the Design of Efficient Fluorescent Probes

Nanotechnologies can benefit in three ways to the design of efficient fluorescent probes dedicated to *in vivo* imaging: (1) by taking benefit of the nanometer-size governed biodistribution of the probe; (2) by designing, in an easy and versatile manner, complex and modular tracers associating different functionalities; (3) by obtaining brighter fluorescent tracers.

**Figure 5:**
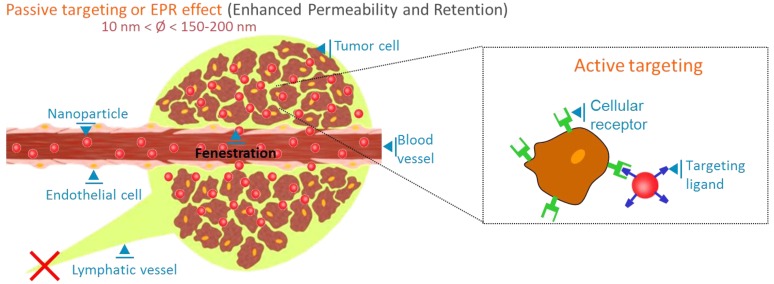
Passive and active tumour targeting of nanoparticles.

It is now well established that nanosized objects can passively accumulate in tumours more efficiently than in healthy tissues. This process, named the “enhanced permeability and retention“ (EPR) effect is linked to the presence of fenestrations (up to a few hundreds of nanometers) in the blood angiogenic vessels, which are built to supply fast-growing tumour cells, associated with the inefficient lymphatic drainage of cancer tissues [46] (Figure 5). This process is not observed for low molecular weight fluorophores (<5 nm), for which fast extravasation from the blood vessels to the tissues is counterbalanced by the inverse diffusion process. Low molecular weight dyes can however be designed to display stronger affinity for tumour than healthy tissues [43]. The passive EPR targeting effect, demonstrated in tumours for a wide range of nanoobjects, could also exist in inflamed tissues and be used for the diagnostics of different pathologies. Passive tumour uptake (generally leading to signal-to-background ratio of about 2:1 to 4:1) can also be enhanced by the decoration of the nanoparticle surface with targeting ligands, able to specifically bind to receptors overexpressed by tumour cells (Figure 5). Different receptors and associated ligands are identified and widely used in animal models, such as folate or vascular endothelial growth factor (VEGF) receptors, and the α_v_β_3_ integrins. If targeted tracers generally yield improved signal-to-background ratios (5:1 or higher) in comparison to EPR effect, as well as molecular information, their signal is intimally linked to the molecular nature of the tumour tissues, and a cocktail of several tracers might be necessary to establish diagnostics. On the contrary, nanoparticles based on EPR effect could constitute “universal” first intention diagnostic contrast agents.

As mentioned earlier, the use of nanoparticle templates also allows the easy design of complex objects. For instance, a nanoparticle surface can be decorated by a controlled number of targeting moieties, promoting cell recognition by the multimerised presentation of the ligands. Drugs or contrast agents for different modalities, including fluorescence imaging,can be associated to design multifunctional probes [47,48] or for theranostic strategies [49,50]. Such objects have been reviewed recently and will not be discussed in the present document [47,48,49,50].

The use of nanostructures is also expected to lead to improved optical properties of the tracer, and ultimately, higher image contrast. Indeed, as will be detailed in the next paragraph, the construction of fluorescent nanotracers can rely either on two different strategies: the use of intrinsically fluorescent semi-conducting nanocrystals (quantum dots); the encapsulation or binding of low molecular weight fluorophores to nanoscale templates (silica or organic nanoparticles for instance). Quantum dots display outstanding optical properties, such as high absorption coefficient and fluorescence quantum yields, but raise cytotoxicity issues. The inclusion of several dyes in a single nanostructure increases their local concentration on the targeted site. Moreover, in the latter dye-inclusion strategy, the nano-matrix in which the fluorophores are encapsulated plays a protecting role, preventing direct chemical contact between the dye and the biological fluids. This notably prevents dye water solubility issues and aggregation, often responsible for loss of optical properties (increased sensitivity to photo-bleaching, protein quenching effects). Therefore, the use of nanotechnologies can lead to the design of more efficient and targeted nanoprobes for high contrast fluorescence imaging.

## 4. Fluorescent Nanoprobes

**Figure 6:**
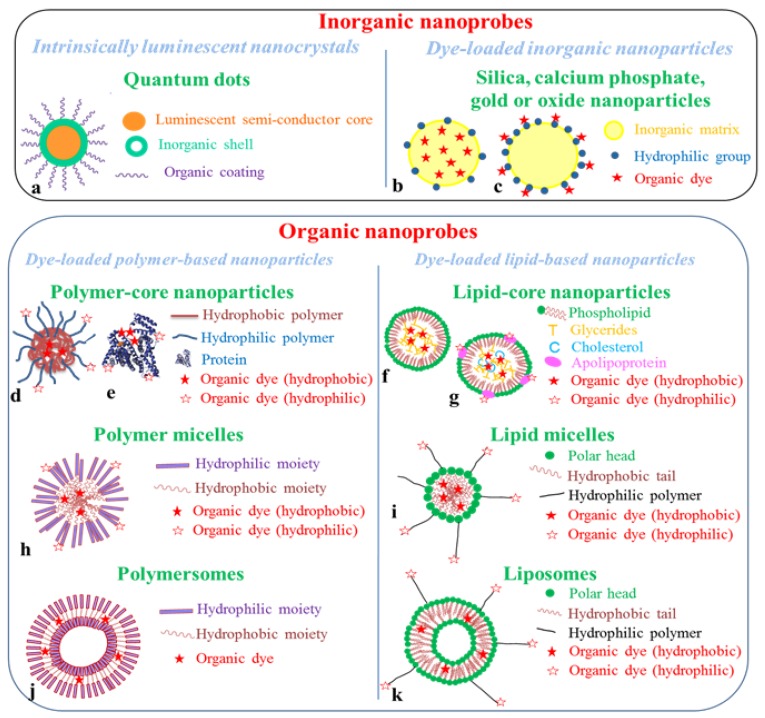
Schematic structures of fluorescent nanoprobes for *in vivo* imaging. Inorganic nanoprobes are quantum dots (**a**) or dye-loaded silica, calcium phosphate, gold or oxide nanoparticles, for which the organic dye can be included in the inorganic matrix (**b**), or grafted on the nanoparticle surface (**c**). Organic nanoprobes can be divided in two main families: dye-loaded polymer-based and dye-loaded lipid-based nanoparticles. In each family, different architectures can be found: polymer- or lipid- core particles (polymer nanospheres (**d**), proteins (**e**), lipid nanoparticles (**f**), lipoproteins (**g**)), self-assembled constructions (polymer (**h**) or lipid (**i**) micelles), nanocapsules (polymersomes (**j**) or liposomes (**k**)). The fluorescent organic dye can be either included in the hydrophobic core or shell of the structure, or grafted on the nanoparticle surface (hydrophilic organic dye).

Fluorescent nanoprobes can be distinguished in two main categories: those based on organic nanoparticles and those constructed with inorganic nanocrystals, such as semi-conducting quantum dots or silica nanoparticles (Figure 6). In this paragraph, the main *in vivo* preclinical studies carried out using fluorescent nanoprobes will be reviewed.

### 4.1. Quantum Dots

Quantum dots are nanocrystals of semi-conducting materials [51]. They are made of elements from the II and VI periodic groups (*i.e.*, ZnS, CdSe or CdTe compositions for instance), or more recently III and V periodic groups (InP) and IV and VI periodic groups (PbS, PbSe, PbTe). They are chemically grown and their emission properties, being related to the confinement effect of an electron-hole pair (exciton) within the crystal, mainly depend on their shape and size. The inorganic semi-conducting core (typically 2–12 nm diameter) is generally coated by an inorganic shell (typicall ZnS), which reinforces the optical properties of the crystal. The outer shell can further be functionalized by an organic layer- typically thiol bi-functional molecules or amphiphilic polymers-, or entrapped in phospholipid micelles (Figure 6a). 

Thanks to their optical properties - tunable, narrow and symmetric emission band, long luminescence lifetime, and good photostability -, these objects have been very intensively studied for bio-imaging since late 1990s [52,53,54,55,56]. Cadmium-free nano-crystals emitting in the near-infrared more specifically dedicated to *in vivo* applications have been developed [57,58,59,60,61,62,63,64]. A decisive step towards their potential use in clinics has been made in 2005 with the evidence that very small neutral nanocrystals (diameter < 5–6 nm) could be eliminated by kidney clearance [65]. This was further confirmed by other studies [62]. Indeed, larger quantum dots are known to accumulate for very long time in reticulo-endothelial system (RES) organs, especially in liver [63,66,67], even if no major acute or prolonged toxicity effects seem to have been observed in rodents [68,69]. The coating of the nanoparticles with poly(ethyleneglycol) (PEG), a well-known polymer to prevent surface protein adsorption and prolong blood lifetime of drugs or nanoparticles [70], does not hinder their RES uptake [71]. Associated to the fact that many quantum dot core compositions include toxic elements such as cadmium, arsenide or lead, it clearly constitutes an obstacle for their translation to clinics. Therefore, even if studies demonstrated the possibility to passively or actively address the particles to tumour sites following their intravenous injections [60,64,72,73], their future use in tumour diagnostics in clinics seems compromised. The application of quantum dots for sentinel lymph node fluorescence mapping [26,61,64,74,75] could be more promising. This application should be less restricting concerning toxicology issues, since a local injection and therefore a reduced dose are required with further removal of the tracer included in the resected node.

### 4.2. Dye-Loaded Inorganic Nanocarriers

Other inorganic probes have been proposed as fluorescent nanotracers, relying on the encapsulation (Figure 6b) or surface binding (Figure 6c) of organic fluorescent dyes to inorganic nanoparticles, mainly silica, calcium phosphate oxide or gold nanoparticles. 

One of the most described silica nanoprobes are certainly the C-dots developed at Cornell University [76], which entered clinical trials in Fall 2011 for lymph node diagnosis and staging in advanced melanoma [77]. Other groups also develop dye-loaded silica nanoparticles for bioimaging applications [78,79,80,81,82]. Silica is an interesting material for the design of functional nano-objects: active molecules can be encapsulated inside the pores of the matrix, or the use of organic modified silanes can allow their covalent attachment in the core or the shell of the particles. Synthesis protocols such as the Stöber ‘s process are well mastered, and easy to implement [83]. The optical properties of the dyes such as cyanines are well preserved and even enhanced in the silica matrix [84,85,86]. Similarly to quantum dots, very small nanocrystals (<10 nm) seem to undergo urinary excretion [81,87], whereas larger nanoparticles (>20 nm) rather distribute in liver, spleen, stomach and undergo hepatobiliary excretion [80]. 

Calcium phosphate (also named apatite) or calcium phosphosilicate have also been proposed as interesting matrixes for dye encapsulation and *in vivo* vectorization [88,89,90,91], but very few preclinical experiments have been performed in rodents [88,89]. 20 nm diameter gold nanoparticles have been used as templates for the covalent grafting of cyanine fluorophores via metalloprotease-cleavable spacers [92]. The cyanine emission is quenched by the surface-energy transfer properties of the nanoparticles, and further activated in protease-rich tissues, such as in SSC7 carcinoma, after breaking of the bound by which they are linked to the gold surface [92]. This design of “activatable probe”, possible thanks to the environment-sensitivity of organic dye fluorescence, allows imaging with high signal-to-background ratio, and underlines one of the benefit that can be brought by fluorescence imaging in comparison to other modalities to get molecular information *in vivo*. Very small gold nanoclusters (2 nm) have also been shown to display luminescent properties by their own, however with emission wavelength below 600 nm, limiting *in vivo* depth imaging [93].

The biodistribution of different oxide based nanocrystals loaded with or encapsulating organic fluorophores have been studied, and is governed, as for other inorganic nanoparticles, by their size, charge, and nature of the polymer coating [94,95]. Yttrium oxide (Y_2_O_3_) [96,97] or sodium yttrium fluoride (NaYF_4_) [75,98,99] nanoparticles can also be loaded with erbium and terbium cations to achieve up-converting nanomaterials. Up-converting nanoparticles are excited in the infrared range (typically 980 nm) and display up-converted emission in the green or near infrared depending on doping concentrations [75,96,97,98,99]. Due to the up-conversion process, tissue autofluorescence is considerably lowered, achieving very high-to-background ratio in biological samples. However, the multiphotonic process involved in up-conversion requires the use of imaging systems with high power excitation light, and imaging is restricted to surface (a very few mm of penetration depth) [75,96,98].

### 4.3. Dye-Loaded Organic Nanocarriers

Organic nanocarriers have aroused much interest for the last 30 years for *in vivo* drug vectorisation, and several nanosized drug formulations are presently used in clinic [100,101]. Therefore, several set of data are already available on the biodistribution and *in vivo* fate of most of these nanocarriers [102,103,104,105,106,107,108,109], even if each nanoparticle displays unique properties. It has well been evidenced that a stealth coating (PEG essentially) is necessary to limit rapid nanoparticle uptake in the RES and allow their distribution in different tissues [110,111,112]. Targeting strategies using different set of ligands such as antibodies, peptides and saccharides have also be explored in different animal models, especially for tumour targeting. In the present review, we will mainly focus on near-infrared dye loading in the organic nanocarriers, its impact on fluorophore properties, and the potential clinical applications that can be envisioned with these systems.

The loading of nanoparticles with organic fluorophores to design dedicated *in vivo* fluorescent nanoprobes has been explored quite recently, mainly in the last 10 years, with the arrival of small animal imaging devices [4,5,6,7]. The fluorescent dyes, mainly near infrared cyanines, porphyrines or phtalocyanines as far as *in vivo* imaging is concerned, can be included in the nanoparticle core, can be intercalated in or be part of the nanoparticle shell, or linked to the particle surface by either adsorption or a covalent bond (conjugation) (Figure 6d–k). Encapsulation, either by the entrapment of the dye using molecular affinity (*i.e.*, a lipophilic dye will present a strong affinity with a lipophilic matrix) or by covalent conjugation to the material constituting the particle core (a hydrophobic polymer chain for instance), presents the advantage of protecting dyes from direct interactions with biological fluids, which can alter their optical properties. Chemical conjugation of the dye to the particle core or shell limits its leakage while in buffered media.

We will divide the organic nanocarriers in two main families, polymer-based and lipid-based nanoparticles (Figure 6). In both categories, “natural” and “synthetic” template nanoparticles are found. For instance natural template nanoparticles include proteins (such as serum albumin, Figure 6e), natural polymers (dextran, chitosan…), bacteriophages or lipoproteins (Figure 6g), whereas synthetic template nanoparticles include polymer nanospheres (Figure 6d), polymer micelles (Figure 6h), dendrimers, liposomes (Figure 6k), solid lipid nanoparticles (Figure 6f).

#### 4.3.1. Polymer-Based Nanoparticles

**Figure 7:**
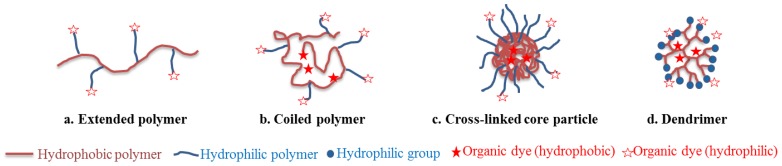
Different architectures of “polymer-core nanoparticles”.

Polymer-based nanoparticles can present different architectures (Figure 6 and Figure 7): linear chains arranged in extended (Figure 7a) or coiled conformation (Figure 7b), according to polymer hydrophobicity, cross-linked core nanospheres (Figure 7c), dendrimers (Figure 7d), which were grouped in Figure 6 as “polymer-core nanoparticles”, self-assembled amphiphilic micelles (Figure 6h), polymersome capsules (*i.e.*, “polymeric” liposomes, Figure 6j) [104]. Polyesters, as well as poly(lactic acid) (PLA) and poly(lactic-*co*-glycolic acid) (PLGA), which hydrophilic/hydrophobic balance can be finely tuned, are extensively used as synthetic nano-materials for their biocompatibility and biodegradability. “Nature-based” polymers include proteins (human serum albumin for instance), polypeptide constructions (poly(lysine), poly(glutamic acid) for example), saccharides (dextran, chitosan).

**Figure 8:**
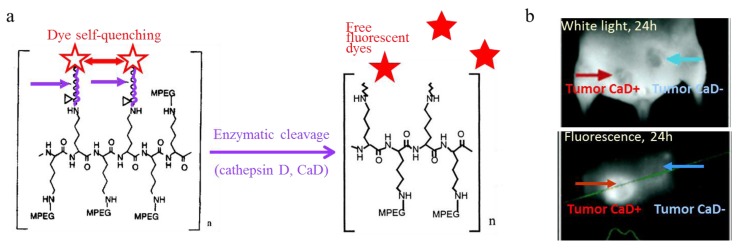
Design of activatable probes for protease activity imaging. (**a**) Architecture of the probe, based on linear poly(lysine) with peptide-fluorophore moieties on pending amino groups. In this example, the peptide sequence can be cleaved by cathepsin D (CaD). (**b**) *In vivo* fluorescence images obtained 24 h after i.v. injection of the reporter probe in Nude mice implanted with a CaD+ (red arrow) and CaD− (blue arrow) tumours. Adaptedfrom Tung, C.-H.; Mahmood, U.; Bredow, S.; Weissleder, R. *In vivo* imaging of proteolytic enzyme activity using a novel molecular reporter. *Cancer Res.* **2000**, *60*, 4953–4958 [116] by permission from the American Association for Cancer Research.

Linear poly(lysine) chains bearing peptide-fluorophore moieties on pending amino groups were one of the first architecture proposed for the design of fluorescent nanoprobes for *in vivo* imaging, and certainly remains one of the most elegant (Figure 8) [113,114,115,116]. The peptide-fluorophore ratio on the polymer backbone was optimized so that the spatial proximity of the fluorophores induced self-quenching of their emission properties. Therefore, the nanoprobe was non-emissive upon injection in the animal tail vein. The peptide sequence linking the fluorophore and the polymer was selected according to the desired proteolytic activity to image. Enzymatic cleavage occured specifically in tissues where the proteases were expressed, freeing the fluorophores, which recovered their emission properties as they diffused in the tissues.

The encapsulation of ICG in different polymer or co-polymer matrixes (typically particle diameter of 100–500 nm) was shown to improve the dye optical properties and enhance its chemical stability [117,118,119,120]. The encapsulation of different dyes in PEG-PLA nanospheres has been studied [121]. The experiments showed that the amphiphilic Nile Red dye (logP = 3.8) was quickly released from the polymer matrix after injection resulting in its rapid elimination from the blood stream, whereas the hydrophobic near infrared DiR dye (logP = 17.4) lead to a constant fluorescent signal in blood up to 6 hours post injection. DiR-loaded particles subsequently allowed tumour labelling [121]. The covalent conjugation of the Cy5 fluorescent dye to the polymer matrix prevented the burst released effect [122]. Such polymer nanoparticles, largely studied as drug delivery systems, are known to be efficiently up taken by the reticulo-endothelial system if not functionalized by a stealth coating such as poly(ethyleneglycol) [110]. 

Near infrared fluorescence dyes were also encapsulated in poly(ethylene imine) or biodegradable aliphatic polyester dendrimers (typically 10–30 nm diameter). The rather small sizes of these polymer structures favour their renal clearance [123]. The dendrimers can be surrounded by a PEG shell to improve their water solubility. The fluorophores thus display enhanced stability, resistance to enzymatic oxidation and prolonged *in vivo* residence time [124]. These structures can also be used to transport both hydrophilic and hydrophobic molecules [125].

The adsorption of ICG on plasma proteins to prolong the otherwise very short blood lifetime of the dye has already been mentioned (see part 2.2) since this constitutes the only clinical trials made with nanosized probes in the field of fluorescence imaging until now [12]. Block co-polymer micelles have also been used to encapsulate ICG [126,127] or other dyes [128]. Polymeric nanomicelles with a PEG shell were functionalized with different targeting ligands for *in vivo* sentinel lymph node mapping or tumour follow-up [129]. Because micelles are dynamic structures in equilibrium with the free monomers at the critical micelle concentration (cmc), the polymer design must be optimized to lower the cmc and avoids fast burst release of the fluorescent dye upon *in vivo* administration.

Bacteriophage particles were functionalized conjointly by pH-sensitive (HCy-646) and pH-insensitive (Cy5) cyanine dyes [130]. This double labelling was used to evaluate the stability of the nanocarrier after injection by double checked in fluorescence signal, and to probe the disruption in acid/base homeostasis often found in tumour hypoxia. 

**Figure 9:**
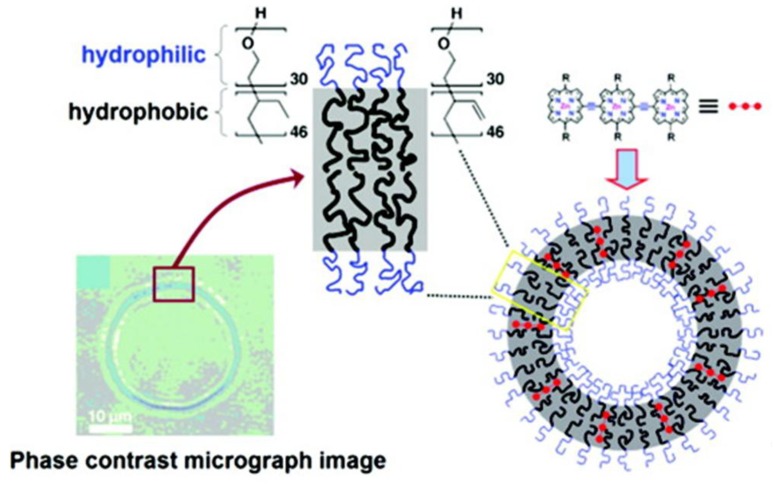
NIR-emissive polymersomes. In aqueous solution, amphiphilic poly(ethylene oxide)/poly(butadiene) polymers (PEO_30_-PBD_46_) self-assemble into polymer vesicles (polymersomes) with the hydrophobic PBD tails oriented end-to-end to form a bilayer membrane, in which (porphynato)zinc(II) oligomers can be encapsulated. Adapted from Duncan, T.V.; Ghoroghchian, P.; Rubtsov, I.V.; Hammer, D.; Therien, M. Ultrafast excited-state dynamics of nanoscale near-infrared emissive polymersomes. *J. Am. Chem. Soc.* **2008**, *130*, 9773–9784 [132]. Copyright (2008) American Chemical Society.

Polymersomes are polymer vesicles generated through cooperative self-assembly of amphiphilic diblock copolymers (similar structure than liposomes but with a polymer instead of a phospholipid bilayer). Their near-infrared properties can be generated including in the polymer shell monomeric or oligomeric dyes (Figure 9) [131,132]. These polymersomes were designed in order to uniformly distribute numerous large hydrophobic porphyrins exclusively in their lamellar membranes. Within these sequestrated compartments, long polymer chains regulated the mean fluorophore-fluorophore interspatial separation as well as the fluorophore-localized electronic environment to optimize optical properties [132,133].

As a conclusion on polymer-based fluorescent nanoprobes, the choice of the matrix polymer and the chemical process (often employing organic solvents whenever synthetic polymers are concerned) is crucial to achieve low-cytotoxicity dye-loaded nanocarriers with good optical properties and suitable biodistribution. Thanks to the well mastered synthetic chemical process, the wide range of materials available, polymer-based probes with finely tuned properties and sizes ranging from 10 nm (dendrimer, micelles) to micrometer (polymer spheres or capsules) can be designed and precisely characterized. Very different biodistribution patterns can be achieved, according to the particle size, charge, and flexibility [123,134], opening the way to design well-tailored specific fluorescent probes for each envisioned medical applications. 

#### 4.3.2. Lipid-Based Nanoparticles

Lipid-based nanoparticles also present a wide variety of architectures, constructed with lipophilic lipids such as mono-, di- or triglycerides, and/or amphiphilic lipids such as phospholipids, which present surfactant properties. The lipids and phospholipids used can be extracted and purified from natural products such as animal or vegetal oils, or synthetically produced. Polymeric moieties, can also be included in the structures: for instance hydrophilic poly(ethyleneglycol) moieties are often grafted on the external bilayer of liposomes to prevent their rapid *in vivo* RES uptake and subsequent degradation. The great advantage of lipid nanocarriers is their intrinsic very low cytotoxicity, which make them outstanding nanocargos concerning potential cytotoxicity issues encountered in clinical applications. For instance, IC_50_ values as high as 0.1 to 1 mg/mL of lipids have been reported for liposomes or solid lipid nanoparticles [135].

**Figure 10:**
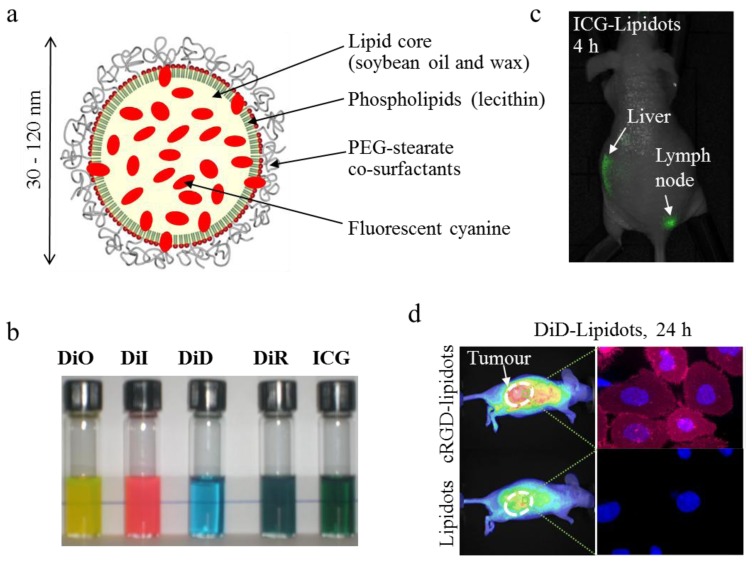
Dye-loaded lipid nanoparticles (“lipidots™”) for *in vivo* imaging. (**a**) Structure of the particles. (**b**) Photograph of 50 nm diameter lipidots loaded with different organic dyes, covering the visible and near-infrared range. (**c**) Lymph node imaging 4 hours after sub-dermal injection of ICG-lipidots in the right paw. (**d**) Specific uptake of cRGD-functionalized lipidots in comparison to non-functionalized nanoparticles in HEKβ3 xenografted tumours in Nude mouse. This tumour model is known for its poor EPR effect and over-expression of α_v_β_3_ integrins, for which the cRGD ligand presents a strong affinity (right images: microscopy photographs of HEKβ3 cells (nuclei stained in blue) after 24 h incubation in the presence of DiD-loaded lipidots (in red)). Adapted from [24,143].

Lipid nanoparticles [105,108,109], nanocapsules [107] or nanoemulsions [106] (particle diameter typically of 50–300 nm) have been studied for a few years for the delivery of lipophilic drugs. It was shown that ICG formulated in soybean emulsion displays improved optical properties and slower plasmatic clearance [136]. Oil nanodroplets encapsulating iron oxide crystals were stabilized by dye-functionalized phospholipids for multimodality imaging [137], whereas perfluorocarbon nanoparticles were loaded with a hydrophobic near-infrared dye for sentinel lymph node mapping using photoacoustic imaging [138]. Our group developed a new technology for the encapsulation of lipophilic molecules, including near infrared dyes, based on oil-in-water nanoemulsions processed by ultrasonication (Figure 10) [139,140]. The main drawback of nanoemulsions -namely intrinsic poor colloidal stability- has been overcome by the use of a complex mixture of core lipids (mixture of long-chain mono-, di- and triglycerides) and surfactants (phospholipids and PEG-stearate), bringing entropy mixing stabilization to the physico-chemical system [140]. These dye-loaded lipid nanoparticles, termed “Lipidots™” display very low cytotoxicity (IC_50_ ≈ 300 µg/mL of lipids [24]), high *in vivo* tolerance dose (>150 mg lipids/kg), and distribute in liver in 30 minutes following their intravenous injection in healthy animals [141]. Their liver uptake seems to be mainly due to the accumulation of the particles in hepatocytes rather than macrophages, since very low levels are detected in spleen and lungs, other organs known for macrophage homing. Lipid nanoparticles can be loaded with different lipophilic dyes [24], including ICG [142], to obtain highly bright fluorescent nanoprobes (Figure 10b). Passive tumour uptake of 50 nm-diameter DiD-loaded Lipidots™ has been demonstrated in a variety of tumour models implanted sub-cutaneous in *Nude* mice using fluorescence imaging [143,144]. The grafting on the nanoparticle surface of the cRGD peptide, exhibiting specific adhesion to α_v_β_3_ integrins overexpressed in 25% of tumour cell lines, can improve tumour accumulation and cell internalization of the functionalized lipid nanoparticles (Figure 10d) [143]. ICG-loaded lipidots also constitute promising nanotracers for sentinel lymph node mapping (Figure 10c) [24,142]. For this later application, we demonstrated that 10 times less DiD-loaded lipidots (2 pmol) could lead to the same image contrast than commercial QTracker™ quantum dots (20 pmol), while displaying decreased cytotoxicity on NIH-3T3 fibroblasts (IC_50_ < 30 nM for quantum dots, >70 nm for DiD-loaded lipidots) [24].

Another strategy is to use as fluorescent dye nanocarriers natural lipoproteins present in the blood stream, especially high density lipoproteins (HDLs) and low density lipoprotein (LDLs) [145,146]. Lipoproteins (10 to 30 nm diameter) are formed of a glyceride and cholesterol core, coated by a phospholipid and apolipoprotein shell (Figure 6g). HDLs [147,148,149] and LDLs [150,151] were modified by the inclusion of fluorescent phospholipids, or lipophilic fluorophores such as DiR or DiR-bis-oleate. The surface of the lipoproteins can be modified to target epidermal growth factor (EGF) or folate receptors, overexpressed in different tumour cells. Moreover, HDLs and LDLs are involved in cholesterol transport and linked to cardio-vascular affections. Dye-loaded apolipoproteins could therefore also constitute interesting nanocarriers for atherosclerosis imaging.

Phospholipid-PEG micelles were shown to improve ICG optical properties and prolong up to a few weeks its stability in aqueous buffer [127,152]. The PEG extremity can also be functionalized to design targeted nanomicelles directed against folate receptors and α_v_β_3_ integrins [127]. However, in a similar way than polymer micelles, lipid micelles must be optimized to lower the cmc and avoids fast burst release of the fluorescent dye upon *in vivo* administration.

Liposomes labelled with the DY-676-C_18_ ester dye were used for near infrared optical imaging of cultured macrophages and of inflammatory process like oedema in an *in vivo* mouse model [153]. ICG was included in different liposomal formulations (typical diameter from 70 to 150 nm) to image tumours [154,155], arthritic tissues [154] or lymphatic vessels [156]. Porphysomes, nanovesicles formed from porphyrine-modified phospholipids self-assembled in bilayers, allowed imaging of the lymphatic system using photoacoustic tomography and fluorescence [157]. 

To conclude on lipid-based fluorescent nanoprobes, specific features seem to be associated to these nanostructures. Indeed, lipid-based nanocarriers have been reported to display strong affinity for lymphatic channels and lymph nodes [23,24,138,142], more importantly than polymeric particles [138]. Their lipid nature could also favor their affinity for numerous tumors over-expressing lipoprotein receptors (breast and prostate cancer especially) [149,150] or atherosclerotic lesions [158]. Another point to underline is their intrinsic very low cytotoxicity, with for instance IC_50_ values as high as 0.1 to 1 mg/mL of lipids for liposomes or solid lipid nanoparticles [135,141]. Taking together, these properties should favor the emergence of dye-loaded lipid-based nanostructures to dedicated imaging applications in clinic. On the contrary, lipid-based nanostructures can appear more difficult to tailor than polymer constructions, which can be very finely tuned for each specific application.

## 5. Conclusions and Perspectives: Transfer to the Clinic

During the last decades, advances in technology (lasers, cameras) have enabled the development of high performance instrumentation for near-infrared fluorescence imaging. This imaging modality has rapidly been propelled toward clinical applications, since the near-infrared region is the best suitable optical window for body in-depth scanning. The use of the currently available near-infrared dyes, especially FDA-approved ICG, has been fully revisited by extending the field of their clinical applications. Due to the depth issue (photon penetration into tissues), near-infrared fluorescence imaging applications have been restricted to superficial tissues, or deep tissues in intraoperative context. For instance, clinical trials are presently ongoing on the accurate identification and resection of the sentinel lymph node, using fluorescence guided surgery. 

In the meantime, nanotechnologies have opened exciting new avenues in the quest of more photostable and highly bright near-infrared tracers, more performing than classical organic dyes in terms of optical properties. The use of nanostructures in comparison to small organic dyes is mainly driven by their different and tunable biodistribution that can be tuned according to article size, shape, charge and flexibility. The wide range of nanostructures explored for the design of efficient nanoprobes is impressive. Quantum dots present several strong physicochemical advantages for this purpose, but their short term translation toward clinics seems impeded by the toxicity issue still in debate for their use in patients. Another encouraging way relies on the entrapment of organic near-infrared dyes into toxic element-free nanoparticles, especially those developed in pharmaceutical science for drug delivery. These safe nanoparticles, especially polymer- or lipid- based carriers, allow the protection of the near-infrared dyes against chemical and/or biological degradation, improve their photophysical properties, and can be targeted for reaching specific diseased cells, increasing thus the fluorescence signal-over-noise ratio. *In fine*, the choice of a fluorescent nanoprobe dedicated to a specific medical application will be discussed on the basis of toxicity/adverse effects at the dose to be injected for good image contrast. The same criterion will dictate the choice between small organic dye and nanometer size tracer. Certainly, several nanoprobes of different chemical nature will come to market for different applications in the near future.

The development of such interesting smart nanoprobes usually relies on interdisciplinary teams in basic sciences in fields ranging from physics to biology, in close environment to clinicians. Their future translation to clinics depends on the hardiness of their production processes in Good Manufacturing Practice (GMP) conditions, their compelling safety, and the benefit they may offer for human health in indicated clinical applications (early diagnosis, functional imaging guided surgery…). Taking all together the aforementioned elements, there is no doubt that new clinical uses of fluorescent nanoprobes will emerge in the next few years. 
